# Robotic Aortic Valve Replacement in Severe Scoliosis with Markedly Narrow Chest

**DOI:** 10.70352/scrj.cr.26-0336

**Published:** 2026-07-22

**Authors:** Sayaka Honda, Naonori Kawamoto, Yuki Tadokoro, Rieko Kutsuzawa, Ayumi Ikuta, Takashi Kakuta, Kota Suzuki, Kizuku Yamashita, Satsuki Fukushima

**Affiliations:** National Cerebral and Cardiovascular Center, Department of Cardiovascular Surgery, Suita, Osaka, Japan

**Keywords:** robotic-assisted aortic valve replacement, chest wall deformity, longitudinal aortotomy

## Abstract

**INTRODUCTION:**

Robotic-assisted aortic valve replacement (AVR) has emerged as an extension of minimally invasive cardiac surgery, offering enhanced visualization and articulated instrument control. However, patients with severe thoracic deformities and markedly narrow chest anatomy are generally considered challenging candidates because restricted operative space may compromise annular exposure, instrument maneuverability, and aortotomy closure. Reports describing robotic-assisted AVR in such patients remain limited. We describe a successful robotic-assisted AVR in a patient with severe scoliosis and a markedly narrow thoracic cavity, highlighting technical modifications that enabled safe valve implantation.

**CASE PRESENTATION:**

A 61-year-old man presented with progressive exertional dyspnea and fatigue caused by severe aortic regurgitation. CT demonstrated severe scoliosis with marked narrowing of the thoracic cavity, resulting in a sternovertebral distance of 6.5 cm. The patient also had a stable chronic type B aortic dissection extending from the descending thoracic aorta to the level of the superior mesenteric artery without involvement of the ascending aorta or aortic arch. After multidisciplinary discussion and informed consent, robotic-assisted AVR was performed using the da Vinci Xi system (Intuitive Surgical, Sunnyvale, CA, USA). Technical modifications included the placement of the service-port robotic arm in the fifth intercostal space to improve the working angle and avoid arm interference, and the creation of a longitudinal aortotomy to facilitate valve exposure in the restricted operative field. Exposure of the noncoronary and left coronary annulus was achieved through robotic retraction and bedside suction assistance. A 23-mm bioprosthetic valve was successfully implanted without intraoperative complications. The patient was extubated within 5 h and discharged on POD 20. Postoperative echocardiography demonstrated normal prosthetic valve function without paravalvular leakage.

**CONCLUSIONS:**

Robotic-assisted AVR may be feasible in carefully selected patients with scoliosis-associated narrow chest anatomy. Detailed preoperative assessment and tailored technical strategies, including longitudinal aortotomy and modified port placement, may facilitate adequate exposure and safe valve implantation in anatomically challenging cases.

## Abbreviations

AVR
: aortic valve replacement
ICS
: intercostal space
MICS
: minimally invasive cardiac surgery
RAVR
: robotic-assisted endoscopic aortic valve replacement
TTE
: transthoracic echocardiography

## INTRODUCTION

Minimally invasive AVR has become an established alternative to median sternotomy. However, patients with severe thoracic deformities or markedly narrow chest anatomy are often considered suboptimal candidates because restricted operative space may limit annular exposure, instrument maneuverability, and aortotomy closure.^1)^

RAVR offers enhanced 3D visualization and articulated instrument motion, potentially overcoming some of these anatomical limitations. Nevertheless, experience with RAVR in patients with severe chest wall deformities remains limited. We report successful RAVR in a patient with severe scoliosis and a markedly narrow thoracic cavity, focusing on technical modifications that enabled safe valve implantation.

## CASE PRESENTATION

A 61-year-old man presented with progressive exertional dyspnea and fatigue. TTE showed severe aortic regurgitation with a preserved left ventricular ejection fraction and left ventricular dilatation (end-systolic diameter, 44 mm; end-diastolic diameter, 63 mm). Mild-to-moderate mitral regurgitation was also present.

The patient was diagnosed with type B aortic dissection 2 months earlier and had been managed conservatively without progression. CT demonstrated a chronic type B aortic dissection extending from just distal to the left subclavian artery to the level of the superior mesenteric artery. The false lumen remained patent without aneurysmal dilatation, malperfusion, or evidence of disease progression. There was no involvement of the ascending aorta or aortic arch.

Enhanced CT demonstrated marked scoliosis with narrowing of the thoracic cavity. The sternovertebral distance was only 6.5 cm (**Fig. 1**). The aortic valve was tricuspid, with an annular diameter of 20.8 × 24.6 mm and normal sinus of Valsalva dimensions.

**Fig. 1:**
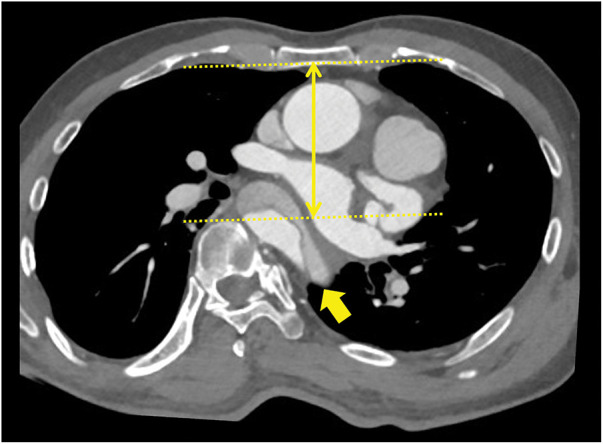
Preoperative CT of the trunk. The yellow arrow indicates a chronic type B aortic dissection. The yellow double-headed arrow outlines the spinal curvature associated with scoliosis. The sternovertebral distance was 6.5 cm.

At the time of referral, the patient’s worsening exertional dyspnea and fatigue were considered primarily attributable to severe aortic regurgitation and left ventricular volume overload.

Although conventional median sternotomy was considered, the patient strongly preferred a minimally invasive approach. Therefore, RAVR using the da Vinci XI robotic system (Intuitive Surgical, Sunnyvale, CA, USA) was selected after careful multidisciplinary discussion and obtaining informed consent from the patient. The operative technique is described below.

The procedure was completed successfully without intraoperative complications. The operation time, cardiopulmonary bypass time, and myocardial ischemic time were 304, 200, and 137 min, respectively. The patient was extubated within 5 h. Follow-up TTE on POD 6 demonstrated a left ventricular ejection fraction of 44% and normal aortic valve function, with no residual paravalvular leakage or transvalvular gradient. The patient was discharged on POD 20 after the recovery of daily living following prolonged rehabilitation, which was required due to a preexisting mobility impairment.

### Surgical technique

General anesthesia was induced with double-lumen endotracheal intubation, and bilateral arterial lines, a central venous catheter, and a Swan–Ganz catheter (Edwards Lifesciences Corp., Irvine, CA, USA) were placed. A right internal jugular venous cannula was inserted before surgical draping.

A 4-cm right anterior mini-thoracotomy was created in the third ICS along the midclavicular line (main incision). A left robotic arm port was inserted through the second ICS along the midclavicular line. The camera port was introduced through the main incision at the right third ICS. A right arm port was placed through the right fourth ICS along the anterior axillary line, and a service port through the right fifth ICS along the midclavicular line (**Fig. 2**). Because of the markedly restricted thoracic cavity, the service-port robotic arm was intentionally positioned in the fifth ICS rather than a more cranial location to avoid arm interference and to improve the working angle toward the aortic annulus. Cardiopulmonary bypass was established via the right subclavian artery, internal jugular vein, and femoral vein.

**Fig. 2:**
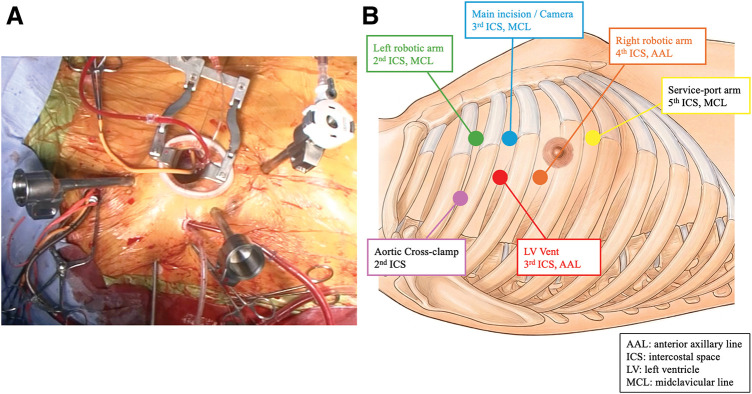
Operative setup and robotic port configuration. (**A**) Intraoperative view of the robotic setup and port configuration. (**B**) Schematic illustration of robotic port placement, left ventricular vent insertion, and Chitwood aortic cross-clamp positioning. AAL, anterior axillary line; ICS, intercostal space; LV, left ventricle; MCL, midclavicular line

A left ventricular vent catheter was inserted through a stab incision in the right third ICS along the anterior axillary line and advanced into the left ventricle via the right upper pulmonary vein. An antegrade cardioplegia cannula was inserted into the ascending aorta through the main incision.

The ascending aorta was cross-clamped using a Chitwood clamp introduced through the second ICS. Cardiac arrest was achieved using antegrade crystalloid cardioplegia. Additional selective antegrade cardioplegia was administered directly into the coronary ostia as needed by the console surgeon using a robotically guided ostial cannula under direct robotic visualization.

The da Vinci Xi robotic system (Intuitive Surgical, Sunnyvale, CA, USA) was docked. A DeBakey forceps was mounted on the left robotic arm, monopolar curved scissors on the right robotic arm, and a Cardiere forceps on the service-port arm. The Cardiere forceps was primarily used for valve and annular retraction to optimize exposure within the restricted thoracic cavity.

Instead of the transverse aortotomy commonly used in conventional and MICS AVR, a straight longitudinal aortotomy was created on the lateral aspect of the ascending aorta (**Fig. 3A**). This approach provided direct visualization of the valve and facilitated subsequent closure despite the limited thoracic dimensions. Exposure of the noncoronary and left coronary annular regions was initially restricted because of the narrow chest and mediastinal displacement. Retraction using the service-port robotic arm improved visualization of the posterior annulus and enabled circumferential suture placement.

**Fig. 3:**
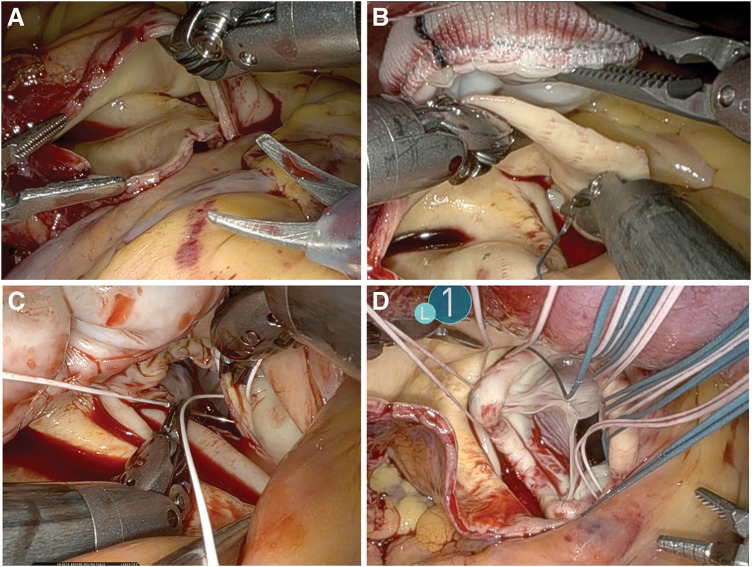
Intraoperative robotic views during robotic-assisted AVR. (**A**) Longitudinal aortotomy performed on the lateral wall of the ascending aorta. (**B**) Manipulation of the prosthetic valve using a Cardiere forceps inserted through the service-port robotic arm. (**C**) Exposure of the aortic annulus using the left and right robotic arms in conjunction with a suction device operated by the bedside surgeon. (**D**) Implantation of the bioprosthetic valve. AVR, aortic valve replacement

The tricuspid aortic valve was completely excised, and after annular sizing, a 23-mm Epic Max bioprosthetic valve (Abbott Laboratories, Chicago, IL, USA) was selected. The prosthetic valve was manipulated within the thoracic cavity using a Cardiere forceps inserted through the service-port robotic arm, allowing stable positioning during valve implantation (**Fig. 3B**). Although exposure of the noncoronary and left coronary annulus was limited by the narrow thoracic cavity, combined retraction using the robotic arms and suction assistance from the bedside surgeon provided adequate visualization for circumferential suture placement (**Fig. 3C**). Fourteen pledgeted 3-0 Ethibond sutures (Ethicon, Raritan, NJ, USA) were placed using a non-everting mattress technique and secured with the COR-KNOT device (LSI Solutions, Victor, NY, USA). The valve was successfully implanted (**Fig. 3D**).

The aortotomy was closed robotically using a double-layer running 5-0 polypropylene suture (Ethicon) technique. After de-airing maneuvers and confirmation of hemostasis, the robotic system was undocked and removed from the operative field. The patient was then weaned from cardiopulmonary bypass. Intraoperative TTE confirmed satisfactory prosthetic valve function without paravalvular leakage.

## DISCUSSION

Thoracic wall deformities can complicate MICS by restricting operative space and altering mediastinal orientation.^1)^ Our patient had severe scoliosis with a markedly narrow thoracic cavity and mediastinal displacement, anatomical features generally considered unfavorable for minimally invasive AVR.

Robotic-assisted cardiac surgery offers distinct advantages in anatomically constrained environments, including high-definition 3D visualization and articulated instruments that facilitate precise intracardiac manipulation within a limited operative field. However, RAVR remains technically demanding and requires careful patient selection and substantial institutional experience. In the present case, adequate annular exposure was achieved through close cooperation between the console surgeon and the bedside surgeon. Furthermore, robotic surgery lacks tactile feedback, which may make assessment of tissue tension and bleeding control more challenging. Therefore, an experienced bedside surgeon remains essential for exposure, suction assistance, and management of unexpected intraoperative events.

A key technical feature of this case was the use of a longitudinal aortotomy. Although transverse aortotomy is commonly used in RAVR,^2)^ a longitudinal aortotomy created a wide opening in the lateral ascending aorta and sinotubular junction directly facing the robotic camera, thereby providing satisfactory visualization of the aortic valve and annulus despite the narrow sternovertebral distance. Nakamizo et al. reported that a longitudinal aortotomy in minimally invasive AVR improves visualization and facilitates safe valve implantation during minimally invasive AVR,^3)^ findings that were consistent with our experience.

Another technical modification was the placement of the service-port robotic arm in the fifth ICS. Because of the markedly restricted thoracic cavity, a more cranial position was anticipated to increase the risk of robotic arm interference. The caudal port position provided a more favorable working configuration and allowed the procedure to be completed without instrument collision.

The patient experienced no intraoperative complications, and postoperative echocardiography demonstrated excellent prosthetic valve function. Although this single case does not establish the general feasibility of RAVR in all patients with thoracic deformity, it suggests that carefully selected patients with severe scoliosis and narrow chest may still be candidates for RAVR when detailed preoperative anatomical assessment and tailored technical modifications are applied.

## CONCLUSIONS

Robotic-assisted AVR may be feasible in selected patients with scoliosis-associated narrow chest anatomy. Careful preoperative assessment and tailored strategies such as longitudinal aortotomy and modified port placement may facilitate adequate surgical exposure and safe valve implantation in anatomically challenging cases.
